# Deletion of Forkhead Box M1 Transcription Factor from Respiratory Epithelial Cells Inhibits Pulmonary Tumorigenesis

**DOI:** 10.1371/journal.pone.0006609

**Published:** 2009-08-12

**Authors:** I-Ching Wang, Lucille Meliton, Xiaomeng Ren, Yufang Zhang, David Balli, Jonathan Snyder, Jeffrey A. Whitsett, Vladimir V. Kalinichenko, Tanya V. Kalin

**Affiliations:** 1 Division of Pulmonary Biology and the Perinatal Institute, Cincinnati Children's Hospital Research Foundation, Cincinnati, Ohio, United States of America; 2 Department of Medicine, University of Chicago, Chicago, Illinois, United States of America; Roswell Park Cancer Institute, United States of America

## Abstract

The Forkhead Box m1 (Foxm1) protein is induced in a majority of human non-small cell lung cancers and its expression is associated with poor prognosis. However, specific requirements for the Foxm1 in each cell type of the cancer lesion remain unknown. The present study provides the first genetic evidence that the Foxm1 expression in respiratory epithelial cells is essential for lung tumorigenesis. Using transgenic mice, we demonstrated that conditional deletion of *Foxm1* from lung epithelial cells (*epFoxm1^−/−^* mice) prior to tumor initiation caused a striking reduction in the number and size of lung tumors, induced by either urethane or 3-methylcholanthrene (MCA)/butylated hydroxytoluene (BHT). Decreased lung tumorigenesis in *epFoxm1^−/−^* mice was associated with diminished proliferation of tumor cells and reduced expression of *Topoisomerase-2α (TOPO-2α)*, a critical regulator of tumor cell proliferation. Depletion of *Foxm1* mRNA in cultured lung adenocarcinoma cells significantly decreased *TOPO-2α* mRNA and protein levels. Moreover, Foxm1 directly bound to and induced transcription of the mouse *TOPO-2α* promoter region, indicating that *TOPO-2α* is a direct target of Foxm1 in lung tumor cells. Finally, we demonstrated that a conditional deletion of Foxm1 in pre-existing lung tumors dramatically reduced tumor growth in the lung. Expression of Foxm1 in respiratory epithelial cells is critical for lung cancer formation and *TOPO-2α* expression *in vivo*, suggesting that Foxm1 is a promising target for anti-tumor therapy.

## Introduction

Lung cancer is the leading cause of cancer-related deaths in men and women in the United States [1]. It has a high mortality because it is difficult to detect early and is frequently resistant to available chemotherapy and radiotherapy. Therefore, identification of proteins regulating the proliferation of lung tumor cells will provide novel targets for diagnosis and treatment of human lung cancer. Lung cancer is classified into small cell lung cancer (SCLC) and non-small cell lung cancer (NSCLC). Adenocarcinoma, the most common type of NSCLC, is frequently associated with gain of function mutations in the K-Ras oncogene [2], [3], activation of c-myc protein, as well as the loss of function mutations in the tumor suppressor gene p53 [4], [5]. Activating mutations in K-Ras also occur in the majority of spontaneous and chemically induced lung tumors in mice [6].

The Foxm1 transcription factor is broadly expressed in actively proliferating cells of all origins [7]. Activation of the Ras-MAPK signaling pathway drives cell cycle progression by regulating the temporal expression of Cyclin regulatory subunits, that activate their corresponding Cyclin-dependent kinases (Cdk) through complex formation. Cdk/cyclin complexes phosphorylate and activate a variety of cell cycle regulatory proteins, including Foxm1 [2], [5], [8]. Activated MAPK (ERK) kinase directly phosphorylates the Foxm1 protein, contributing to its transcriptional activation [9]. Foxm1 directly stimulates the transcription of genes essential for progression into DNA replication and mitosis, including *cyclin B1*, *Cdc25B phosphatase*, *Aurora B kinase* and *Polo-like kinase 1* [10]. *Foxm1* ^−/−^ mice exhibit embryonic lethality due to severe proliferation defects in the developing heart, liver, and blood vessels [11].

Consistent with an important role of Foxm1 in cell cycle progression, increased expression of Foxm1 was found in human lung adenocarcinomas and squamous cell carcinomas, prostate adenocarcinomas, basal cell carcinomas, intrahepatic cholangiocarcinomas, anaplastic astrocytomas and glioblastomas, infiltrating ductal breast carcinomas, as well as in many other solid tumors (reviewed in [10], [12], [13], [14]). Inhibition of Foxm1 in cultured tumor cells with either siRNA transfection or pharmacological agents caused a cell cycle arrest [15], [16], [17], [18], [19]. Previous studies with Mx-Cre *Foxm1^fl/fl^* mice demonstrated that deletion of Foxm1 from all cell types of the body caused a significant reduction in number and size of lung adenomas induced by urethane [20]. Furthermore, over-expression of Foxm1 in all cell types in Rosa26-Foxm1 transgenic mice significantly increased the number and size of lung tumors induced by MCA/BHT lung tumor induction/promotion protocol [21]. Although these studies demonstrated a critical role of Foxm1 in lung tumorigenesis, specific requirements for this transcription factor in different populations of respiratory cells remain unknown. Since lung cancer lesions contain a heterogeneous population of cells, that includes tumor cells, originated from genetically modified epithelial cells, and different inflammatory and stromal cells [22], it is important to know the cell autonomous role of Foxm1 during lung tumorigenesis. In the present study, transgenic mice were generated in which the *Foxm1* gene was conditionally deleted in lung epithelial cells (*epFoxm1^−/−^* mice) either prior to the initiation of chemically-induced lung cancer, or during cancer progression/expansion. Deletion of Foxm1 from epithelial cells caused a striking decrease in the number and size of lung adenomas. Decreased lung tumorigenesis in *epFoxm1^−/−^* mice was associated with diminished proliferation of tumor cells and reduced expression of *Topoisomerase-2α (TOPO-2α)*, a critical regulator of tumor cell proliferation. Depletion of *Foxm1* mRNA in cultured lung adenocarcinoma cells significantly decreased *TOPO-2α* mRNA and protein. Foxm1 directly bound to and induced transcription of the mouse *TOPO-2α* promoter region, indicating that *TOPO-2α* is a direct transcriptional target of Foxm1. Taken together, our data demonstrate that Foxm1 expression in respiratory epithelial cells is critical for expansion of lung cancer and TOPO-2α expression in lung tumor cells.

## Results

### Conditional deletion of Foxm1 transcription factor in lung epithelial cells

**Figure 1:**
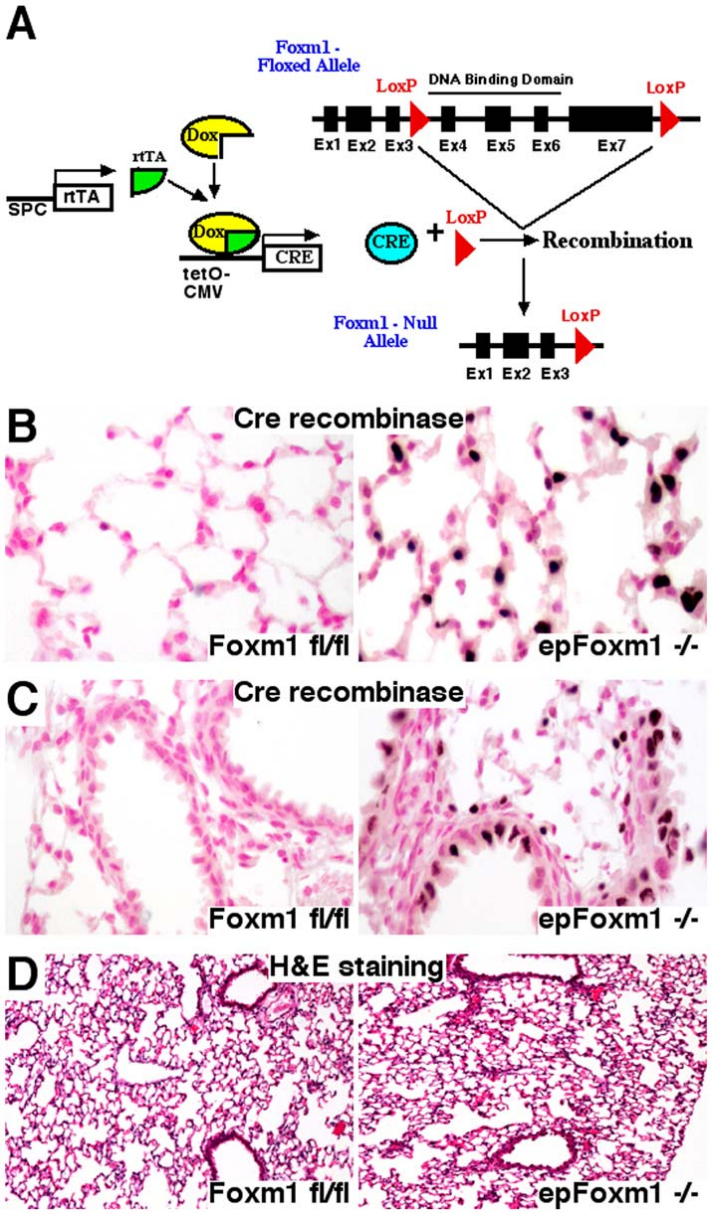
Conditional deletion of Foxm1 transcription factor in lung epithelial cells. A. Breeding strategy for conditional expression of rtTA in the respiratory epithelium to delete the *Foxm1* floxed exons *in vivo*. Dox-induced expression of Cre-recombinase causes the deletion of exon 4–7 of Foxm1 gene (*epFoxm1^−/−^* mice). B–C. Specific expression of Cre in lung epithelium. Lungs from *epFoxm1^−/−^* and control *Foxm1* ^fl/fl^ mice, that were given Dox in water for 4 weeks, were fixed, paraffin-embedded, sectioned, and stained with Cre antibody (dark brown nuclei) and then counterstained with nuclear fast red (red nuclei). Cre expression was observed in type II lung epithelial cells (B) and Clara cells (C), but not in mesenchymal cells of Dox-treated *epFoxm1^−/−^* lungs. Cre recombinase was not detected in control *Foxm1^fl/fl^* lungs. D. Conditional expression of Cre in lung epithelial cells does not alter the lung morphology. Paraffin sections from Dox-treated *epFoxm1^−/−^* and control *Foxm1^fl/fl^* lungs were stained with hematoxylin and eosin (H&E). Normal lung alveoli and bronchi were observed in control *Foxm1* ^fl/fl^ and *epFoxm1^−/−^* mice. Magnifications: ×200 (B–C) and ×50 (D).

To determine the role of Foxm1 in epithelial cells during formation of lung tumors, we generated triple-transgenic mice containing LoxP-flanked exons 4–7 of the *Foxm1* gene (*Foxm1* ^fl/fl^), the *SP-C–rtTA* ^tg/−^ and the *TetO-Cre* ^tg/−^ transgenes (*SP-C–rtTA* ^tg/−^ */TetO-Cre* ^tg/−^/*Foxm1* ^fl/fl^ or *epFoxm1* ^fl/fl^ mice). To induce Cre expression in lung epithelial cells, Dox was given to the adult mice in water for 4 weeks. Previous studies demonstrated that using this protocol, Cre-mediated recombination occurs in type II epithelial cells and Clara cells of the adult lung [23]. In the presence of Dox, the reverse tetracycline transactivator (rtTA) binds to the TetO promoter and induces expression of Cre recombinase, deleting exons 4–7, which encode DNA binding and transcriptional activation domains of the Foxm1 protein (Fig. 1A); thus, lung *ep*ithelial-specific *Foxm1* knockout mice (*epFoxm1* ^−/−^) are generated. Consistent with previous studies, Cre protein was detected in epithelial type II (Fig. 1B) and Clara cells (Fig. 1C) of Dox-treated *epFoxm1* ^−/−^ mice but not in control *Foxm1* ^fl/fl^ mice (Fig. 1B–C). No morphological changes were observed in either *epFoxm1* ^−/−^ or control *Foxm1* ^fl/fl^ lungs after 4 weeks of Dox treatment (Fig. 1D). Thus, the Foxm1 deletion from adult lungs does not alter lung structure.

### Foxm1 deletion from respiratory epithelial cells reduces numbers and sizes of lung tumors

**Figure 2:**
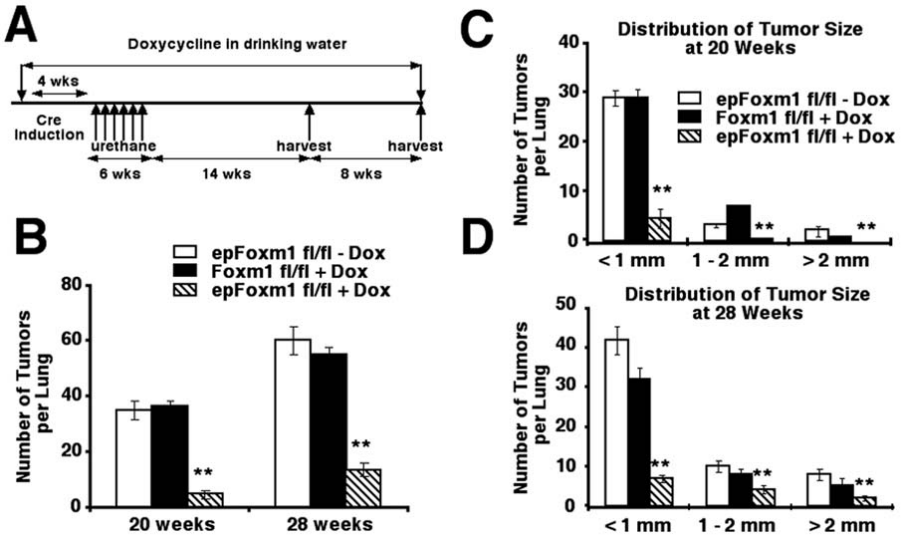
Foxm1 deletion from lung epithelial cells prior to tumor initiation reduced the number and size of urethane-induced lung tumors. Experimental *epFoxm1^−/−^* (*epFoxm1^fl/fl^* +Dox) and control *Foxm1^fl/fl^* (*Foxm1^fl/fl^* +Dox) mice were given Dox in water to activate Cre expression prior to induction of lung tumors by multiple urethane injections as described in Materials and Methods. The second control group of *epFoxm1^fl/fl^* mice was given regular water (*epFoxm1* ^fl/fl^ – Dox; this mouse group was used as a control for Dox-independent recombination). All three groups of mice received six weekly injections of urethane to induce lung tumors. A. Experimental design of conditional deletion of *Foxm1^fl/fl^* prior to tumor induction. B. Lung tumors from experimental *epFoxm1^−/−^* (*epFoxm1^fl/fl^* +Dox) mice and control mice were counted and measured using a dissecting microscope at 20 weeks and 28 weeks after the first urethane injection. Deletion of *Foxm1* prior to tumor induction significantly reduced (p<0.05) the total number of lung tumors at both time points. C–D. The tumor diameters were significantly reduced (p<0.05) at 20 weeks (C) and 28 weeks (D) after tumor induction. Mean number of tumors per lung (±SD) and tumor sizes were calculated from n = 12 mouse lungs per group.

To determine the role of Foxm1 in epithelial cells during formation of lung tumors, experimental *epFoxm1^fl/fl^* and control male mice were given Dox for 4 weeks. Control groups included Dox-treated *Foxm1* ^fl/fl^ male mice and *epFoxm1^fl/+^*mice, as well as *epFoxm1^fl/fl^* transgenic mice without Dox treatment. Mice were subjected to 6 weekly urethane injections to induce lung tumors (Fig. 2A). Dox treatment was continued for additional 20 or 28 weeks after the first urethane injection. In this model, deletion of Foxm1 from lung epithelial cells was induced prior to the initiation of lung tumorigenesis.

**Figure 3:**
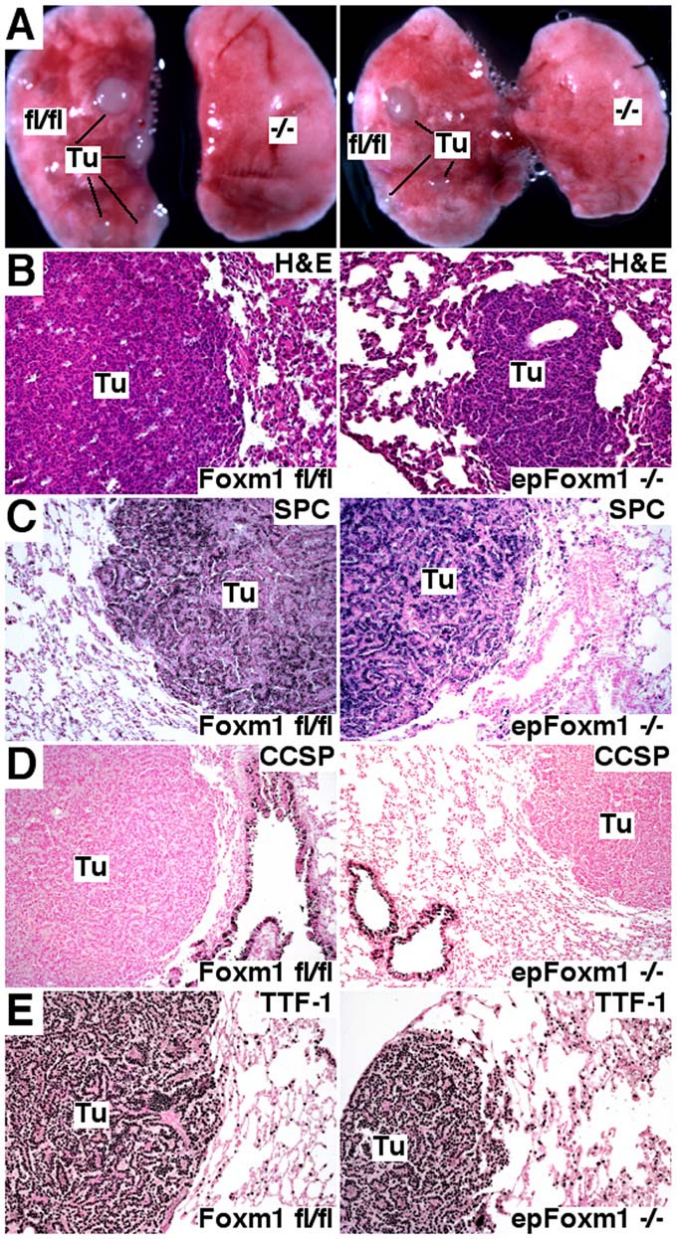
Lung tumors are originated from type II lung epithelial cells. Lungs from *epFoxm1^−/−^* and control *Foxm1* ^fl/fl^ mice, that were given Dox in water starting 4 weeks before urethane injections, were photographed and fixed 28 weeks after the first urethane injection. Lung sections were used either for H&E staining or immunohistochemistry. A. Photographs of control *Foxm1^fl/fl^* mouse lungs (left lungs) depict lung tumors 28 weeks after the first urethane injection. Experimental *epFoxm1^−/−^* mice were resistant to tumor induction (right lungs), showing no visible tumors. B. H&E staining demonstrates a reduction in the size of lung tumors (Tu) in *epFoxm1^−/−^* mouse lungs. C. Lung tumors in control and *epFoxm1^−/−^* are positive for SPC, a marker of type II lung epithelial cells. D. Lung tumors in control and *epFoxm1^−/−^* are negative for CCSP, a marker of Clara cells. E. Similar expression of TTF-1 transcription factor in *epFoxm1^−/−^* and control *Foxm1* ^fl/fl^ tumors. Magnification: A panels, 10×; B–E panels, 100×.

The total number of urethane-induced lung tumors was decreased approximately 5-fold in *epFoxm1^−/−^* (*epFoxm1^fl/fl^*+Dox) mice compared to all control groups (Fig. 2B and data not shown). Numbers of large (>2 mm), medium (1–2 mm) and small-sized (<1 mm) tumors were significantly decreased in *epFoxm1^−/−^* mice at both 20 and 28 weeks after the first urethane injection (Fig. 2C–D and Fig. 3A). Histological examination of H&E-stained sections confirmed the reduced size of lung tumors from *epFoxm1^−/−^* mice and showed that these tumors displayed morphological characteristics of lung adenomas (Fig. 3B). To identify the origin of the lung tumor cells, the immunohistochemical staining was performed using antibodies against either surfactant protein C (SPC), a type II lung alveolar epithelial cell marker, or Clara cell specific protein (CCSP), Clara cells marker. All tumors in control and *epFoxm1^−/−^* mice were SPC- positive (Fig. 3C), indicating that they developed from alveolar type II epithelial cells. No CCSP- positive tumors were found in either *epFoxm1^−/−^* or control *epFoxm1^fl/fl^* lungs (Fig. 3D). Interestingly, *epFoxm1^−/−^* tumors maintained normal expression levels of TTF-1, a lung epithelial-specific transcription factor, implicated in controlling cellular proliferation during embryogenesis and formation of non-small cell lung cancer [24].

### Foxm1 deletion from respiratory epithelial cells diminishes tumor cell proliferation

**Figure 4:**
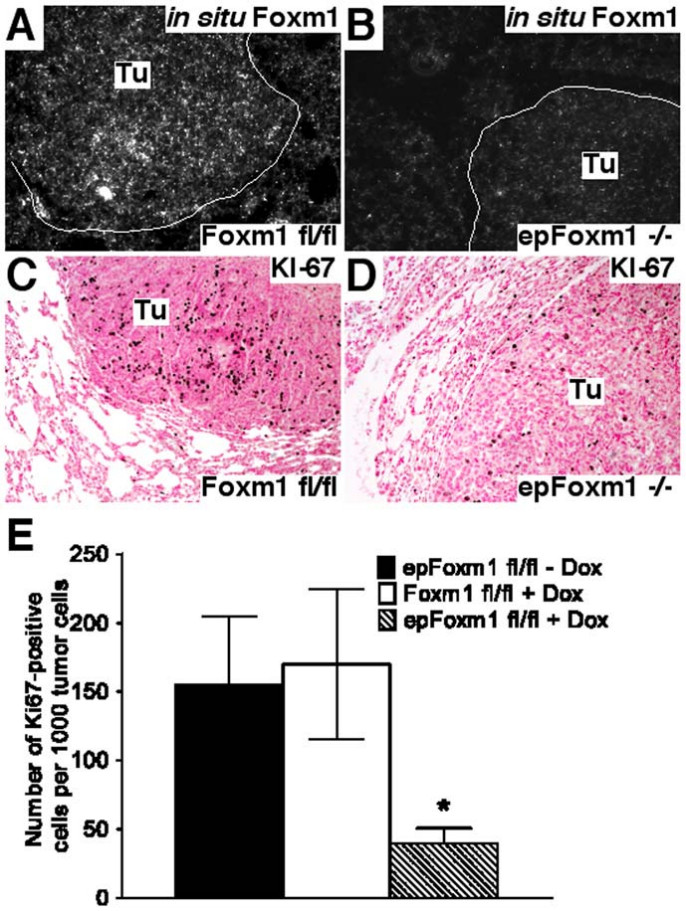
Foxm1 deletion from respiratory epithelial cells diminishes tumor cell proliferation. Lungs from *epFoxm1^−/−^* and control *Foxm1* ^fl/fl^ mice, that were given Dox in water starting 4 weeks before urethane injections, were photographed and fixed 28 weeks after the first urethane injection. Lung sections were used either for *in situ* hybridization or immunohistochemistry. A–B. *In situ* hybridization demonstrates that *Foxm1* mRNA is selectively decreased in lung tumors of *epFoxm1^−/−^* lungs (B) versus control *epFoxm1^fl/fl^* mice (A). C–D. Diminished cell proliferation in *epFoxm1^−/−^* lung tumors (D) is shown using Ki-67 antibody. E. Decreased number of Ki-67-positive cells in *epFoxm1^−/−^* mouse lungs. Ki-67-positive cells were counted in ten random microscope fields from tumor regions of control and *epFoxm1^−/−^* lungs (mean+s.d.). Magnification: A panels, 10×; B–D panels, 100×.

To address the efficiency of deletion of the *Foxm1* ^fl/fl^ allele from lung tumors, *Foxm1* mRNA was examined by *in situ* hybridization of lung paraffin sections. Consistent with efficient Cre-mediated recombination, *Foxm1* mRNA was decreased in lung tumors from *epFoxm1^−/−^* mice compared to *Foxm1* ^fl/fl^ mice (Fig. 4A and 4B). Furthermore, numbers of KI-67 positive cells were significantly reduced in *epFoxm1^−/−^* tumors (Fig. 4C–D and 4E). We did not observe any differences in the number of apoptotic cells in the tumors from control and *epFoxm1^−/−^* mice (data not shown). Altogether, these results indicate that Foxm1 deletion from type II lung epithelial cells is sufficient to reduce proliferation of tumor cells *in vivo* and decrease urethane-mediated lung tumorigenesis.

### Tumors from *epFoxm1^−/−^* mice demonstrate altered expression of genes important for cellular proliferation and lung tumorigenesis

**Figure 5:**
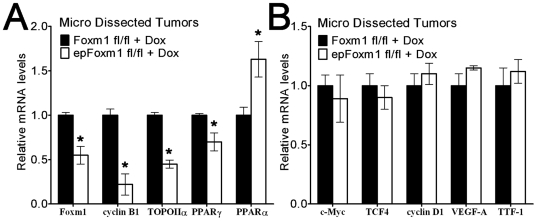
Deletion of Foxm1 influenced the expression of several genes. Microdissected tumors from control *Foxm1^fl/fl^* +Dox or experimental *epFoxm1^fl/fl^* +Dox mouse lungs (n = 3 mice per group) were used to prepare total RNA for Quantitative real-time RT-PCR. A. Decreased *Foxm1*, *TOPO-2α*, *cyclin B1*, *PPARγ* and increased *PPARα* mRNAs were observed in *epFoxm1^−/−^* (*epFoxm1^fl/fl^* +Dox) lung tumors. B. No changes were found in the expression of the *c-Myc*, *TCF-4*, *cyclin D1*, *TTF-1* and *VEGF-A* genes. β-actin mRNA was used for normalization. A *p* value<0.05 is shown with asterisk (*).

To identify new molecular targets of Foxm1 in respiratory epithelial cells, lung tumors were dissected from control and urethane-treated mice and then used to prepare total RNA. Even with the contribution of non-tumor cell RNA (endothelial cells, macrophages, fibroblasts), the Real-time RT-PCR analysis revealed decreased Foxm1 mRNA levels in *epFoxm1^−/−^* tumors compared to control *Foxm1* ^fl/fl^ tumors (Fig. 5A). Foxm1 deficiency in *epFoxm1^−/−^* tumors was associated with decreased mRNA levels of *cyclin B1* (Fig. 5A), a known transcriptional target for Foxm1 [25]. Expression of several genes important for cellular proliferation and lung carcinogenesis was also studied, including *c-Myc*, *cyclin D1*, *TOPO-2α*, *TCF4*, *VEGF-A*, *TTF-1*, *PPARγ* and *PPARα*. While *TOPO-2α* and *PPARγ* mRNA were decreased in lung tumors from *epFoxm1^−/−^* mice, *PPARα* mRNAs was significantly increased (Fig. 5A). No changes were found in the mRNA expression of *c-Myc*, *cyclin D1*, *TCF4*, *TTF-1* and *VEGF-A* genes (Fig. 5B).

### Foxm1 deletion after tumor initiation decreases lung tumor expansion/progression

**Figure 6:**
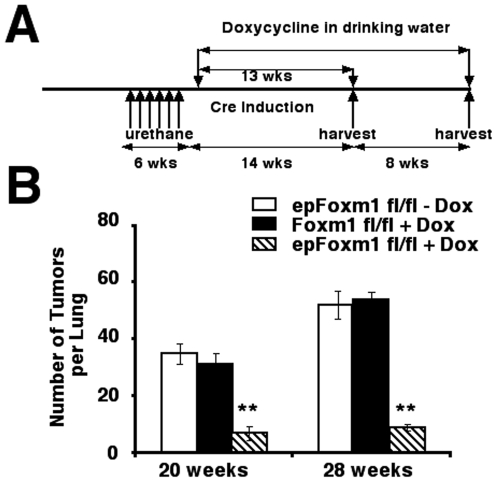
Foxm1 deletion after tumor initiation decreases lung tumor expansion/progression. Experimental *epFoxm1^−/−^* (*epFoxm1^fl/fl^* +Dox) and control *Foxm1^fl/fl^* (*Foxm1^fl/fl^* +Dox) mice were given Dox in water to activate Cre expression after the initiation of lung tumors by multiple urethane injections as described in Materials and Methods. The second control group of *epFoxm1^fl/fl^* mice was given regular water (*epFoxm1* ^fl/fl^ - Dox). All three groups of mice received six weekly injections of urethane to induce lung tumors. A. Experimental design of conditional deletion of *Foxm1^fl/fl^* after tumor induction. B. Lung tumors from experimental *epFoxm1^−/−^* (*epFoxm1^fl/fl^* +Dox) mice and control mice were counted and measured using a dissecting microscope at 20 weeks and 28 weeks after the first urethane injection. Deletion of *Foxm1* after tumor initiation significantly reduced (p<0.05) the total number of lung tumors at both time points.

Having established that deletion of Foxm1 from lung epithelial cells prior to tumor initiation significantly reduced lung tumorigenesis, the next series of experiments were designed to determine whether Foxm1 was required during progression/expansion of lung tumors *in vivo*. Lung tumors in *epFoxm1^fl/fl^* and control *Foxm1^fl/fl^* mice were induced by six urethane injections. One week after the last urethane injection, Dox-treatment was initiated and continued until the completion of the experiment at 20 or 28 weeks (Fig. 6A). Thus, Foxm1 deletion occurs after initiation of lung tumors. A significant decrease in the number of lung tumors was observed in Dox-treated *epFoxm1^−/−^* mice compared to either Dox-treated *Foxm1^fl/fl^* mice or *epFoxm1^fl/fl^* mice without Dox treatment (Fig. 6B). These results demonstrate that Foxm1 expression in the pulmonary epithelium is required for the progression/expansion of urethane-induced lung cancer.

### Foxm1 is essential for lung tumorigenesis induced by MCA/BHT

**Figure 7:**
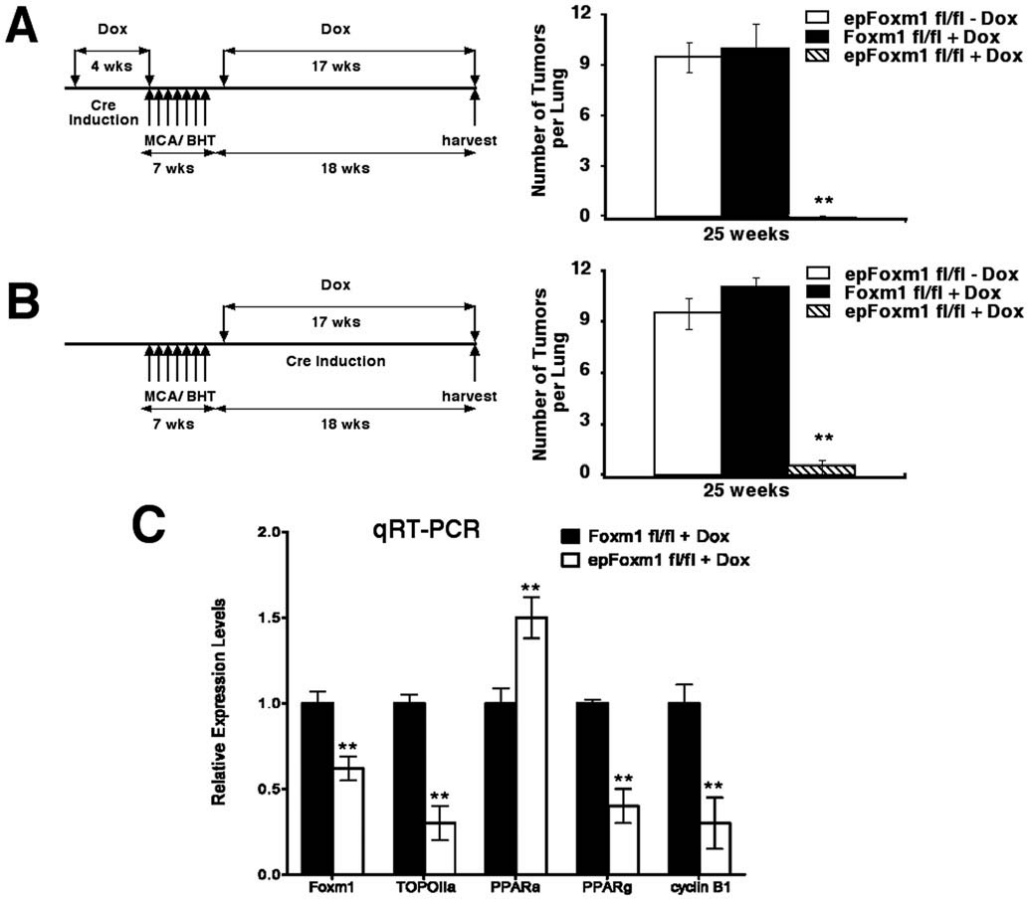
Foxm1 is essential for lung tumor growth induced by MCA/BHT. Experimental *epFoxm1^−/−^* (*epFoxm1^fl/fl^* +Dox) and control *Foxm1^fl/fl^* (*Foxm1^fl/fl^* +Dox) mice were given Dox in water to activate Cre expression either 4 weeks prior to or 1 week after tumor induction/promotion with MCA/BHT as described in Materials and Methods. The *epFoxm1^fl/fl^* (*epFoxm1^fl/fl^* – Dox) mice given regular water were used as controls for possible Dox-independent recombination. All three groups of mice were injected with a single dose of MCA, followed by six weekly injections of BHT to induce lung tumors. Mice were sacrificed at 25 weeks after MCA injection and examined for lung tumors using a dissecting microscope. A. Experimental design for conditional deletion of *Foxm1^fl/fl^* prior to tumor induction/promotion (left panel). *epFoxm1^−/−^* mice are resistant to lung tumorigenesis after MCA/BHT treatment compared to control mice (right panel). Mean number of tumors per lung (±SD) was calculated from n = 10 mouse lungs per group. B. Experimental design for conditional deletion of *Foxm1^fl/fl^* after tumor induction/promotion. *epFoxm1^−/−^* mice are resistant to lung tumorigenesis after MCA/BHT treatment compared to control mice (right panel). Mean number of tumors per lung (±SD) was calculated from n = 10 mouse lungs per group. C. Decreased levels of *Foxm1*, *TOPO-2α*, *PPARγ* and *cyclin B1* mRNAs and increased levels of *PPARα* in *epFoxm1^−/−^* (*epFoxm1* ^fl/fl^+Dox) lung. qRT-PCR was performed using total RNA from control *Foxm1* ^fl/fl^ and *epFoxm1^−/−^* lungs. Three mice per group were used. Asterisks **indicate P values<0.001 calculated by Student T Test.

**Figure 8:**
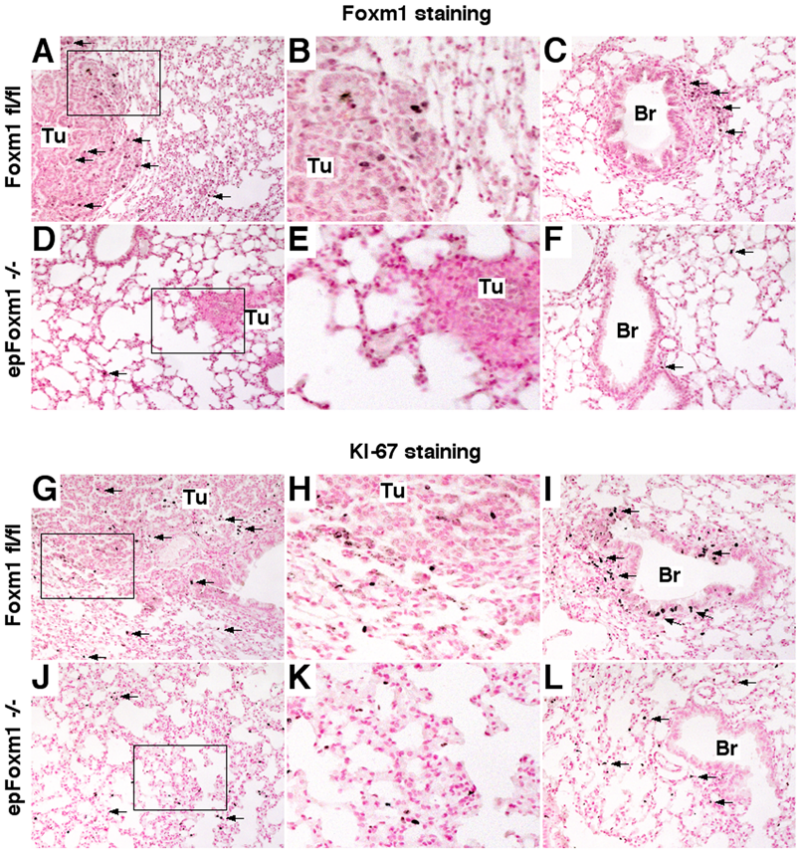
*epFoxm1^−/−^* tumors displayed diminished Foxm1 staining and decreased proliferation after MCA/BHT induced tumorigenesis. Lungs from *epFoxm1^−/−^* and control *Foxm1* ^fl/fl^ mice, that were given Dox in water starting 4 weeks before MCA/BHT treatment, were dissected and fixed 25 weeks after the first injection. Lung sections were used for immunohistochemistry with either Foxm1 (A–F) or KI-67 antibodies (G–L). Nuclear Foxm1 staining (shown with arrows) was detected in a subset of tumor cells (Tu in A–B) and epithelial cells of peribronchial regions of *Foxm1* ^fl/fl^ lungs (C). Although Foxm1 protein was not detected in *epFoxm1^−/−^* tumors, Foxm1-positive epithelial cells were still found in alveolar region of *epFoxm1^−/−^* lungs (arrows in D and F). Diminished KI-67 staining was observed in *epFoxm1^−/−^* lungs (J–L) when compared to control *Foxm1* ^fl/fl^ lungs (G–I). Magnification is 100×. B, E, H and K are inserts from A, D, G and J.

To demonstrate that the established critical role of Foxm1 in lung epithelial cells is not limited only to urethane mediated tumorigenesis, we used another experimental mouse model of lung cancer: two-step lung tumor initiation/promotion using 3-methylcholanthrene (MCA)/butylated hydroxytoluene (BHT). Lung tumors in *epFoxm1^−/−^* and control male mice were induced by a single injection of MCA, a carcinogen found in tobacco smoke [26], [27]. Starting one week after MCA injection, mice were subjected to weekly injections of BHT for 6 consecutive weeks to promote formation of lung tumors by causing chronic pulmonary inflammation and remodeling of the lung [26]. To induce Foxm1 deletion prior to tumor initiation, Dox was administered for 4 weeks prior to MCA injection (Fig. 7A). To avoid negative effects of Dox on lung inflammation, we did not administer Dox to mice during MCA/BHT injections. In another series of experiments, Dox was given one week after BHT treatment, causing Foxm1 deletion after tumor initiation (Fig. 7B). Both types of experiments demonstrated that *epFoxm1^−/−^* mice were resistant to lung tumorigenesis as demonstarated by comparison of tumor numbers in Foxm1-deficient mice to either control *Foxm1* ^fl/fl^ mice, or triple transgenic *epFoxm1^fl/fl^* mice without Dox treatment (Fig. 7A and 7B). The *epFoxm1^−/−^* lungs displayed only few microscopic tumor lesions. Immunohistochemistry demonstrated that decreased numbers and size of lung tumors in *epFoxm1^−/−^* lungs were associated with reduced Foxm1 expression (Fig. 8A–F) and decreased proliferation in tumor regions (Fig. 8G–L). Consistent with urethane experiments, MCA/BHT treated *epFoxm1^−/−^* lungs displayed decreased *TOPO-2α*, *cyclin B1* and *PPARγ* mRNAs, confirming a critical role of Foxm1 in the regulation of these genes in lung tumor cells *in vivo* (Fig. 7C). Altogether, our results demonstrate that Foxm1 in epithelial cells plays a critical role during chemically-induced and inflammation-mediated lung tumorigenesis.

### *TOPO-2α* is a direct target of Foxm1 transcriptional factor in lung epithelial cells

**Figure 9:**
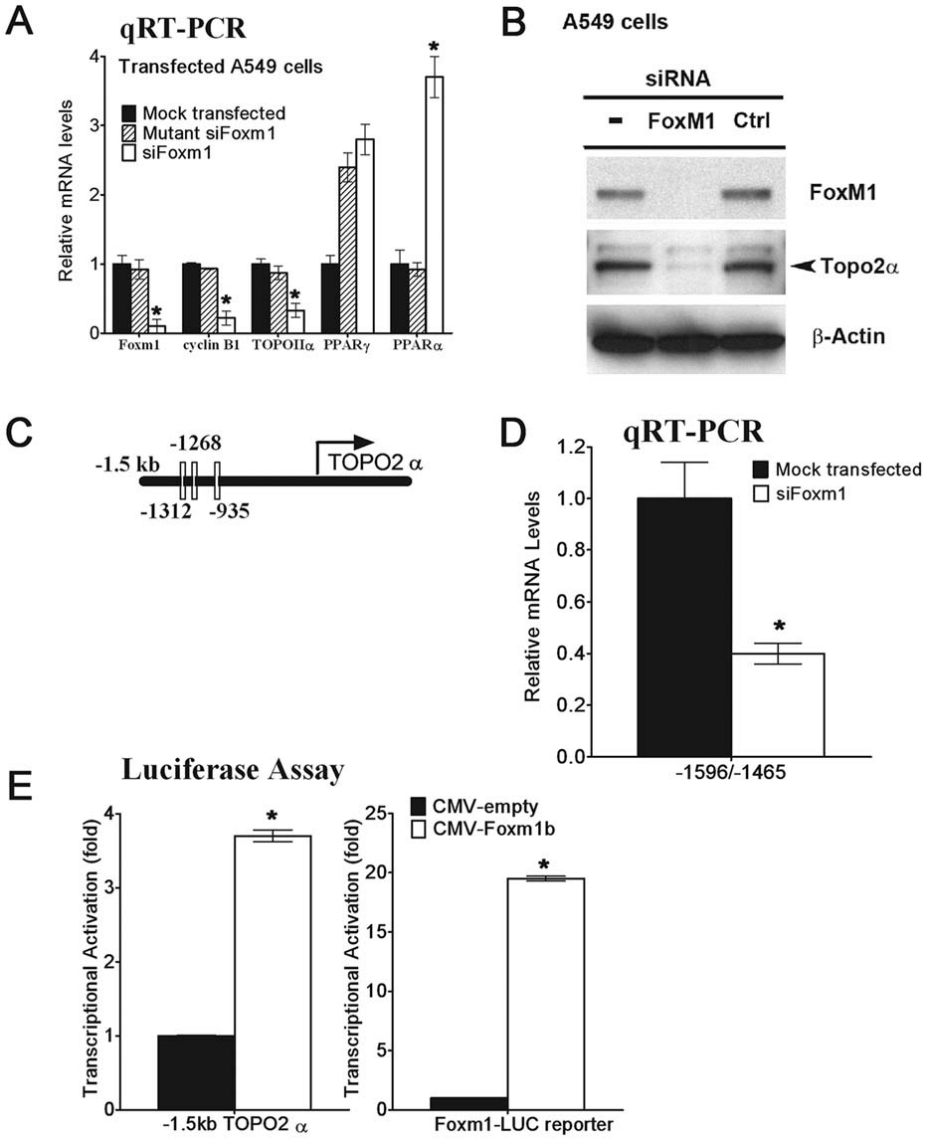
Foxm1 transcription factor directly binds to and induces the mouse *TOPO-2α* promoter region. A. *Foxm1* mRNA depletion in A549 lung adenocarcinoma cells led to decreased *TOPO-2α* and *cyclin B1* mRNAs expression. A549 cells were mock transfected (control) or transfected with short interfering RNA (siRNA) duplex specific for *Foxm1* mRNA (siFoxm1) or with control mutant siFoxm1 duplex. Forty-eight hours after siRNA transfection, total RNA was extracted and analyzed for *Foxm1*, *TOPO-2α*, *PPARα*, *PPARγ* and *cyclin B1* mRNAs by qRT-PCR. B. Western blot shows decreased protein levels of TOPO-2α and Foxm1 in siRNA-transfected A549 cells. C. A schematic drawing of the –1.5 Kb promoter region of the mouse TOPO-2α gene. Locations of three potential Foxm1 DNA binding sites are indicated (white boxes). D. ChIP assay demonstrated that Foxm1 protein binds to promoter regions of the *TOPO-2α* gene. Foxm1 binding to genomic DNA was normalized to IgG control antibodies. Diminished binding of Foxm1 to the endogenous mouse promoter regions of the *TOPO-2α* gene was observed after siFoxm1 transfection in MLE-15 cells. E. Foxm1 induced the transcriptional activity of *TOPO-2α* promoter. MLE-15 cells were transfected with CMV-Foxm1b expression vector and luciferase (LUC) reporter driven by the -1.5 kb mouse *TOPO-2α* promoter region. CMV-empty plasmid was used as a negative control. Cotransfection of CMV-Foxm1b and Foxm1-Luc reporter plasmids were used as a positive control. Cells were harvested at 24 hr after transfection and processed for dual LUC assays to determine LUC activity. Transcriptional activity of the mouse *TOPO-2α* promoter was increased by CMV-Foxm1b transfection. A *p* value<0.05 is shown with asterisk (*).

Our *in vivo* experiments suggested that Foxm1 regulated lung epithelial genes essential for lung cancer formation. To determine whether Foxm1 regulates expression of these genes *in vitro*, A549 human lung adenocarcinoma cells were transfected with short interfering RNA (siRNA) specific to the human *Foxm1* mRNA (siFoxm1) or with mutant control siFoxm1 [13]. Forty-eight hours after siRNA transfection, total RNA was prepared from the A549 cells and analyzed for *Foxm1* expression by qRT-PCR. siFoxm1 transfection efficiently reduced *Foxm1* mRNA, also inhibiting the expression of Foxm1-target *cyclin B1* gene (Fig. 9A). Consistent with our *in vivo* studies (Fig. 5A), Foxm1-depletion *in vitro* significantly decreased *TOPO-2α* mRNA, whereas *PPARα* mRNA was increased (Fig. 9A). Furthermore, protein levels of TOPO-2α were decreased after Foxm1 depletion in A549 cells as demonstrated by Western blot analysis (Fig. 9B).

Lung epithelial-specific ablation of *Foxm1* both *in vivo* and *in vitro* resulted in significant decrease in *TOPO-2α* mRNA expression, which suggests that Foxm1 plays direct or indirect role in the regulation of *TOPO-2α* gene expression. Since TOPO-2α is a prominent target for anti-tumor therapy [28] due to its critical role in tumor cell proliferation, we focused our next experiments to determine whether *TOPO-2α* gene is a direct transcriptional target of Foxm1. Three potential Foxm1 protein binding sites were identified in the –1.5 Kb promoter region of the mouse *TOPO-2α* gene (Fig. 9C). We next used Chromatin Immunoprecipitation (ChIP) assays to determine whether Foxm1 protein directly binds to the -1.5 Kb *TOPO-2α* promoter region in the context of endogenous DNA. The cross-linked and sonicated chromatin from untransfected mouse lung epithelial 15 (MLE-15) cells or MLE-15 cells transfected with Foxm1-specific siRNA was immunoprecipitated (IP) either with antibodies specific to Foxm1 or with IgG control antibodies. Binding of *TOPO-2α* promoter DNA associated with the IP chromatin was determined by qRT-PCR with primers specific to the potential Foxm1-binding region in mouse *TOPO-2α* promoter. Foxm1 protein specifically bound to the *TOPO-2α* promoter region as demonstrated by the ability of siFoxm1 to reduce binding of Foxm1 protein to *TOPO-2α* promoter DNA (Fig. 9D). To determine whether the Foxm1-binding sites were transcriptionally active, co-transfection experiments were performed using CMV-Foxm1b expression vector [29] and luciferase (LUC) reporter construct driven by *TOPO-2α* promoter region. Co-transfection of the CMV-Foxm1b expression vector significantly increased expression of the –1.5 Kb *TOPO-2α* reporter plasmid when compared to CMV-empty vector (Fig. 9E), indicating that Foxm1 is a transcriptional activator of *TOPO-2α* gene. These results demonstrate that Foxm1 directly binds to and transcriptionally activates the mouse *TOPO-2α* promoter region, indicating that *TOPO-2α* is a direct Foxm1 target gene.

## Discussion

Existing treatments for lung cancer have not significantly improved survival, leading to a critical need for new approaches. The important role of Foxm1 in lung cancer has already been established. However, our basic understanding of its role in different cell populations of the tumor is limited. Furthermore, the molecular mechanisms whereby cell autonomous Foxm1 expression regulates lung tumorigenesis remain to be established.

The important contribution of the current study is the establishment of the critical role of Foxm1 in respiratory epithelial cells during formation of lung cancer. The conditional deletion of Foxm1 from lung epithelial cells strikingly decreased the development of lung tumors in mice, diminished proliferation of tumor cells and reduced the expression of *cyclin B1* and *TOPO-2α*. Foxm1 deficiency was also associated with increased expression of *PPAR-α*. Altogether, these observations provide new insight into a cell autonomous role of Foxm1 in the pathogenesis of pulmonary adenocarcinoma, and identify the Foxm1 transcription factor as a potential target for treatment of human lung cancer.

Increased Foxm1 levels were found in numerous types of human tumors, including non small cell lung cancer [14], [20]. Our previous studies demonstrated that when the *Foxm ^fl/fl^* allele was deleted in all cell types using Mx-Cre transgene, the numbers and sizes of lung adenomas following urethane exposure were reduced [20]. On the contrary, over-expression of Foxm1 in all cell types using Rosa 26 promoter led to striking increase in the numbers and sizes of lung tumors induced with MCA/BHT in transgenic mice [21]. Thus, previous gain-of-function or loss-of-function studies with ubiquitous changes in Foxm1 expression had identified Foxm1 as an important transcription factor during lung tumorigenesis. However, lung cancer lesions contain a heterogeneous population of cells, that includes epithelial, inflammatory (macrophages, granulocytes) and stromal cells, that express increased levels of Foxm1 [22] and may influence tumorigenesis. Although our previous studies emphasized the essential role of Foxm1 in lung tumorigenesis, its specific role in lung epithelial cells, the precursors of lung adenoma or adenocarcinoma cells, was not addressed. In the present study, deletion of Foxm1 from respiratory epithelial cells was sufficient to significantly decrease lung tumor formation, demonstrating the cell autonomous role of Foxm1 in the progression of pulmonary tumors.

Two different models of lung cancer in mice were used. In the first model, urethane acts as a complete carcinogen leading to DNA damage with K-ras mutations and providing both initiation and promotion of tumorigenesis [6]. In the second model, MCA, a carcinogen found in tobacco smoke [26], acts only as an initiator (DNA damage), while BHT, acts as a tumor promoter by causing chronic pulmonary inflammation due to necrosis of type I epithelial cells and macrophage infiltration. Both models showed that Foxm1 is critical for lung tumorigenesis. Consistent with these findings, the expression of Foxm1 in colon epithelial cells [30] and hepatocytes [18] was essential for progression of colon cancer and hepatocellular carcinoma, respectively. Moreover, the present data suggest that the main role of Foxm1 in epithelial cells occurs during tumor progression/expansion. We found a similar decrease in lung tumorigenesis regardless if Foxm1 was deleted prior to the tumor initiation or during tumor progression. Therefore, Foxm1 is critical for the proliferation of tumor cells during the expansion of lung tumors.

Since lung tumors were still found in urethane-treated *epFoxm1^−/−^* mice, our results suggest that a subset of lung tumor cells can proliferate in the absence of Foxm1. Interestingly, the *epFoxm1^−/−^* tumors still maintained normal expression levels of the TTF-1 protein, a lung epithelial-specific transcription factor, implicated in controlling cellular proliferation during embryogenesis and formation of non-small cell lung cancer [24]. Published studies demonstrated that increased TTF-1 expression and amplification of *TTF-1* gene occurred in many cases of NSCLC in human patients [31], [32]. We also found that *epFoxm1^−/−^* tumors displayed normal expression levels of the cell cycle promoting c-Myc and Cyclin D1. Our results suggest that TTF-1, c-Myc and Cyclin D1 can contribute to maintaining the low proliferation rates in *epFoxm1^−/−^* tumors. Alternatively, it is also possible that the formation of lung tumors in *epFoxm1^−/−^* mice may result from secondary mutations that allowed tumor formation, bypassing proliferation defects in Foxm1-deficient lung tumor cells.

The present study demonstrated that *TOPO-2α* was decreased after deletion of Foxm1 during pulmonary tumorigenesis and it was shown that Foxm1 directly induces the *TOPO-2α* promoter activity. Type II topoisomerases are ubiquitous enzymes that play essential roles in regulating DNA under- and over-winding, and resolving knots and tangles in the genetic material through transient double-stranded breaks [33]. Because TOPO-2α generates DNA strand breaks, it has the potential to fragment the genome every time it functions and to cause significant genotoxic damage. TOPO-2α levels increase during the cell cycle, peaking in G2/M [34], in close correlation with the expression levels of Foxm1 transcription factor [7]. In the present study, we established that *TOPO-2α* expression was significantly decreased in Foxm1 deficient lung tumors as well as in A549 human lung adenocarcinoma cells transfected *in vitro* with Foxm1-specific siRNA. Chromatin immunoprecipitation assay demonstrated that Foxm1 protein directly binds to the mouse TOPO-2α promoter, suggesting that Foxm1 is a direct transcriptional activator of *TOPO-2α* gene. This is an important finding that can be used for designing the new therapeutic strategies to treat lung cancer.

Deletion of Foxm1 in lung epithelial cells increased *PPARα* expression during tumorigenesis *in vivo*. *PPARα* mRNA was also increased in Foxm1-depleted A549 lung adenocarcinoma cells *in vitro*. PPARα is the member of the nuclear hormone receptor superfamily that acts as a ligand-activated transcription factor. Recent studies indicate that *PPARα* expression is increased in mouse or human medullablastoma cells, leading to the gradual accumulation of cells in G1 and G2/M phases of the cell cycle and inhibition of cell proliferation [35]. Since accumulation of cells in G1 and G2/M and inhibition of cell proliferation are main characteristics of Foxm1 deficiency [25], this recent data is consistent with our findings that increased *PPARα* mRNA in Foxm1 deficient tumors correlated with inhibition of cell prolifetartion. The other study showed that incidence of DEHP-induced hepatocellular carcinomas in PPARα-null mice was significantly higher than in wild type mice [36], suggesting negative relationship between PPARα expression and cell proliferation. Therefore, increased *PPARα* expression in Foxm1-deficient epithelial cells may contribute to reduced tumorigenesis in *epFoxm1^−/−^* mice.

In summary, deletion of Foxm1 in respiratory epithelial cells prior to or even after tumor initiation caused a striking decrease in the number and size of lung tumors. Decreased tumor formation in *epFoxm1^−/−^* lungs was associated with diminished proliferation of tumor cells. Micro-dissected tumors from *epFoxm1^−/−^* lungs showed significant decrease in *TOPO-2α* mRNA expression. Foxm1 induced *TOPO-2α* expression in A549 lung adenocarcinoma cells and directly bound to the *TOPO-2α* promoter region. The present data demonstrates that Foxm1 expression in respiratory epithelial cells is required for progression/expansion of chemically-induced lung cancer *in vivo* and provides support for the concept that Foxm1 functions in a cell autonomous manner during pulmonary carcinogenesis. Our studies suggest that Foxm1 represents a potential therapeutic target in treatment of NSCLC lung cancers.

## Materials and Methods

### Transgenic mice

The generation of *Foxm1^flox/flox^* (*Foxm1^fl/fl^*) mice was described previously [11]. The *Foxm1^fl/fl^* mice were bred with *SPC–rtTA^tg/−^*/*TetO-Cre^tg/tg^* mice [23] to generate the *SPC–rtTA^tg/−^/TetO-Cre^tg/−^/Foxm1^fl/fl^* triple transgenic mice (*epFoxm1* ^fl/fl^). Only male mice were used for tumor studies. To induce Cre expression in respiratory *epithelium* and produce *epFoxm1* ^−/−^ mice, doxycycline (Dox; 1% in drinking water) was given to 8 week old mice. Four weeks later, lung tumors were induced by urethane or MCA/BHT. Dox treatment was continued through the whole experiment (tumor induction and progression studies). In another series of experiments Dox was given starting 1 week after the last urethane or BHT injections until the day of tissue harvest (tumor progression studies). Dox-treated *Foxm1^fl/fl^* male littermates lacking either the *SPC–rtTA*, the *TetO-Cre* or both transgenes were used as controls. Further controls included Dox-treated *SPC–rtTA* ^tg/−^/*TetO-Cre* ^tg/−^/*Foxm1* ^fl/+^ male mice and *SPC–rtTA* ^tg/−^/*TetO-Cre* ^tg/−^/*Foxm1* ^fl/fl^ male mice without Dox treatment (*epFoxm1* ^fl/fl^ – Dox group). Animal studies were reviewed and approved by the Animal Care and Use Committee of Cincinnati Children's Hospital Research Foundation.

### Tumor induction protocols

#### Urethane protocol

Control and *epFoxm1^−/−^* male mice were injected i.p. with 1 mg/g of body weight of urethane (Sigma, St. Louis, MO; diluted in saline) once a week for 6 consecutive weeks and tumors were counted and examined 20 and 28 weeks after the initial injection using a dissecting microscope. Individually microdissected lung tumors were used to prepare total RNA with RNA-STAT-60 (Tel-Test “B” Inc. Friendswood, TX). Lung tissues were fixed and embedded in paraffin blocks.

#### MCA/BHT protocol

MCA (Sigma, St. Louis, MO), a polycyclic aromatic hydrocarbon found in tobacco smoke which serves as a tumor initiator, was given as a single i.p. dose of 15 µg/g of body weight, followed by six weekly i.p. injections with BHT, a known tumor promoter, (200 µg/g of body weight; Sigma, St. Louis, MO). MCA and BHT were solubilized in corn oil. Mice were sacrificed at 25 weeks following MCA injection and examined for lung tumors using a dissecting microscope.

### Immunohistochemical staining and *in situ* hybridization

Paraffin (5 µm) sections were stained with hematoxylin and eosin (H&E) for morphological examination or immunostained with antibodies against KI-67 (1∶500; clone Tec-3, Dako), Cre (1∶30,000; no. 69050–3, Novagen), SPC (1;1,500; AB-3428, Chemicon International), CCSP (1∶1,000; a gift from B. Stripp, Duke University), TTF-1 (1∶3,000, gift of Dr. DiLauro, Stazione Zoologica, Naples, Italy) or Foxm1 (1∶2000; clone K-19; Santa Cruze Biotechnology, Santa Cruze, CA). Antibody-antigen complexes were detected using biotinylated secondary antibody followed by avidin-horseradish peroxidase (HRP) complex, and DAB substrate (all from Vector Lab, Burlingame, CA) as described [21]. Sections were counterstained with nuclear fast red (Vector Labs, Burlingame, CA). Lung sections were also used for *in situ* hybridization with 35S-labeled antisense riboprobe specific to 1649–1947 bp region of the mouse Foxm1 mRNA as described [7].

### Quantitative real-time RT-PCR (qRT-PCR)

Total RNA was prepared from microdissected lung tumors of *epFoxm1* ^−/−^ or control *Foxm1* ^fl/fl^ mice, or from A549 human lung adenocarcinoma cells (purchased from ATCC, Manassas, VA) and analyzed by qRT-PCR using the StepOnePlus Real-Time PCR system (Applied Biosystems, Foster City, CA). Samples were amplified with Taqman Gene Expression Master Mix (Applied Biosystems) combined with inventoried Taqman gene expression assays: mouse Foxm1, Mm00514924_m1; mouse cyclinB1, Mm00838401_g1; mouse PPARα, Mm00440939_m1; mouse PPARγ, Mm00440945_m1; mouse TOPO-2α, Mm00495703_m1; mouse β-Actin, Mm00607939_s1; mouse c-Myc, Mm00487804_m1; mouse TCF-4, Mm00501505_m1; mouse Cyclin D1, Mm00432359_m1; mouse Vegfa, Mm00437304_m1; mouse TTF-1, Mm00447558_m1; human FoxM1, Hs00133543_m1; human Cyclin B1, Hs00259126_m1; human PPARα, Hs00231982_m1; human PPARγ, Hs00234592_m1; human TOPO-2α, Hs01032127_g1; human β–Actin, Hs99999903_m1. Reactions were analyzed in triplicates and expression levels were normalized to β-Actin.

### siRNA transfection

We transfected 100 nmol/L of either Foxm1-specific siRNA (siFoxm1; Dharmacon Research, Lafayette, CO) or mutant control siFoxm1 into human A549 lung adenocarcinoma cells (purchased from ATCC) using Lipofectamine^TM^ 2000 reagent (Invitrogen) in serum free tissue culture media as described previously [13], [20]. Forty-eight hours after transfection, the A549 cells were used to prepare total RNA.

### Western blot

Nuclear protein extracts were prepared from A549 adenocarcinoma cell lines at 48 hours following siRNA transfection or mock transfection using Nuclear/Cytosol Fractionation kit (K266-100, BioVision, Mountain View, CA) following protocols provided by the manufacturer, and Western blot analysis was done as described previously [13]. The rabbit anti-Foxm1 antibody (1∶1000; clone C-20; Santa Cruze Biotechnology, Santa Cruze, CA) and mouse anti-TOPO-2α antibody (1∶2500; cat# MAB4197; Chemicon) were used. The signals from the primary antibody were amplified by HRP-conjugated anti-mouse IgG (Bio-Rad, Hercules, CA) and detected with Enhanced Chemiluminescence Plus (Amersham Pharmacia Biotech, Piscataway, NJ) followed by autoradiography.

### Cotransfection studies and chromatin immunoprecipitation (ChIP) assay

We used PCR of mouse genomic DNA to amplify the -1544/+17 region of the mouse *TOPO-2α* promoter (Gene Bank Number NT_165773.2) using following primers: 5′-GGG GTA C**C**C CCC CCC AAA AAA AAA AAC ACC-3′ and 5′-CGA CGC GTA AAG CGA CAA AAC CAG CGG-3′. The PCR product was cloned into pGL3-Basic firefly luciferase (LUC) reporter plasmid (Promega) and then verified by DNA sequencing (Gene Bank Number NT_165773.2). We transfected mouse lung epithelial MLE-15 cells [37] with either CMV-Foxm1b (Gene Bank number NM_008021) or control CMV-empty expression plasmids, as well as with LUC reporters driven by –1.5 kb mouse *TOPO-2α* promoter. CMV-Renilla was used as an internal control to normalize transfection efficiency. Dual luciferase assay (Promega) was performed 24 hours after transfection as described [37].

Untransfected or siRNA-transfected mouse lung epithelial MLE-15 cells [37] were cross-linked by addition of formaldehyde, sonicated and used for the immunoprecipitation with Foxm1 rabbit polyclonal antibodies (H-300, Santa Cruz, CA) as described previously [25]. DNA fragments were 500 bp – 1000 bp. Reverse cross-linked ChIP DNA samples were subjected to qRT-PCR using the oligonucleotides specific to -1596/-1465 bp region of mouse TOPO-2α (5′- ACTGCCAGGACTACACAGAGGAAC-3′ and 5′-GGAGGGTGACTTCAATGCTTAGG -3′). DNA binding was normalized to control ChIP DNA samples, which were immunoprecipitated using control rabbit serum.

### Statistical analysis

We used Microsoft Excel Program to calculate SD and statistically significant differences between samples using the Student T Test. P values<0.05 were considered statistically significant.
